# Effectiveness of four types of neuraminidase inhibitors approved in Japan for the treatment of influenza

**DOI:** 10.1371/journal.pone.0224683

**Published:** 2019-11-07

**Authors:** Momoko Mawatari, Reiko Saito, Akinobu Hibino, Hiroki Kondo, Ren Yagami, Takashi Odagiri, Ikumi Tanabe, Yugo Shobugawa, on behalf of Japanese Influenza Collaborative Study Group

**Affiliations:** 1 Division of International Health (Public Health), Graduate School of Medical and Dental Sciences, Niigata University, Niigata, Japan; 2 Division of Infectious Diseases and Immunology, Department of Microbiology, School of Medicine, Iwate Medical University, Iwate, Japan; University of South Dakota, UNITED STATES

## Abstract

**Background:**

Neuraminidase inhibitors (NAIs) effectively treat influenza. The clinical effectiveness of four NAIs (oseltamivir, zanamivir, laninamivir, and peramivir) was evaluated against influenza A/H1N1pdm09, A/H3N2, and B viruses. Additionally, fever duration in patients infected with oseltamivir-resistant influenza A/H1N1pdm09 with the H275Y mutation was evaluated.

**Methods:**

Patients aged <20 years who visited outpatient clinics in Japan with influenza-like illnesses were enrolled during 4 influenza seasons from 2012/2013 to 2015/2016. After obtaining informed consent, patients who tested positive for influenza with rapid tests received one of the four NAIs. Patients recorded their body temperature daily for 8 days from the first visit. The influenza strain was identified using real-time polymerase chain reaction. Univariate and multivariable analyses were used to evaluate factors influencing fever duration. In children aged ≤5 years treated with oseltamivir, fever duration in oseltamivir-resistant A/H1N1pdm09-infected patients was compared to that in oseltamivir-sensitive A/H1N1pdm09-infected patients.

**Results:**

Of the 1,368 patients analyzed, 297 (21.7%), 683 (49.9%), and 388 (28.4%) were infected with influenza A/H1N1pdm09, A/H3N2, and B, respectively. In multivariable analysis factors associated with significantly prolonged fever duration included: treatment with laninamivir (hazard ratio [HR]: 0.78, p = 0.006, compared to oseltamivir), influenza B (HR: 0.58, p<0.001, compared to influenza A/H1N1pdm09), and a higher body temperature at the clinic visit (HR: 0.87 per degree Celsius, p<0.001). Increasing age was associated with a significantly shorter duration of fever (HR: 1.31 for 6–9 years old, p<0.001; and HR: 1.65 for 10–19 years old, p<0.001, respectively, compared to 0–5 years old). Following treatment with oseltamivir, fever duration was significantly longer for oseltamivir-resistant A/H1N1pdm09-infected patients (n = 5) than for oseltamivir-sensitive A/H1N1pdm09 infected patients (n = 111) (mean, 89 versus 40 hours, p<0.001).

**Conclusions:**

Our results revealed characteristic information on the effectiveness of the four NAIs and also on oseltamivir-resistant viruses that may affect patients’ clinical care.

## Introduction

Influenza causes morbidity and mortality in humans worldwide, with high socioeconomic burden. In Japan, it is estimated that 10–15 million people are infected with influenza, which is equivalent to more than 10% of the total population [1]. Neuraminidase inhibitors (NAIs) are effective for the prevention and treatment of influenza. The four NAIs, oseltamivir (Tamiflu^®^, Chugai Pharmaceutical Co., Ltd., Tokyo, Japan), zanamivir (Relenza^®^, GlaxoSmithKline plc, London, United Kingdom), laninamivir (Inavir^®^, Daiichi Sankyo Co., Ltd., Tokyo, Japan), and peramivir (Rapiacta^®^, Shionogi & Co., Ltd., Osaka, Japan) are available for clinical use in Japan [2]. These NAIs are prescribed to seven to eight million influenza-infected outpatients and inpatients annually [2].

Neuraminidase is an enzyme on the surface of the virus that is needed to release progeny virions from the host cells [3]. NAIs inhibit the viral neuraminidase, resulting in the blockage of viral replication and transmission. Past reports showed that in patients who received oseltamivir for influenza infections, fever was alleviated approximately one day earlier than in those who did not [4–6]. Similar effect of zanamivir was also reported [7, 8]. Laninamivir and peramivir are exclusively used in Japan; therefore, information on these drugs is limited [2]. The outstanding feature in Japan is that the four drugs can be prescribed to outpatients who are covered by health insurance [2]. Thus, clinicians can decide which drugs to prescribe depending on the patient’s situation and consent. Another peculiar situation is that, oseltamivir has not been recommended for teenagers’ use since 2005 until 2018 due to suspected associations with abnormal behaviors [2].

In 2007–2008, the spread of influenza A/H1N1, which has an amino acid mutation at position 275 histidine to tyrosine (H275Y) in neuraminidase protein (which confers resistance to oseltamivir and peramivir), occurred globally [9]. Patients who were infected with these viruses had prolonged fever following treatment with oseltamivir [10–14]. However, after the circulation of influenza A/H1N1pdm09 began in 2009, only the sporadic outbreaks of oseltamivir-resistance viruses were reported [2, 9, 15–17]. Therefore, limited reports are available regarding the clinical outcome of H275Y mutated A/H1N1 pdm09 virus [18, 19].

In the present study, we evaluated various factors influencing fever duration in patients who received one of the four NAIs against laboratory confirmed influenza infections, at outpatient clinics. Moreover, we evaluated the effectiveness of oseltamivir on H275Y mutated A/H1N1pdm09 virus compared to sensitive viruses.

## Materials and methods

### Study design and patients

An observational study was conducted in 8 clinics in 6 prefectures (Niigata, Gunma, Tokyo, Chiba, Kyoto, and Nagasaki) in Japan through 4 influenza seasons, from 2012–2013 to 2015–2016. Patients, otherwise healthy, aged 0 to 19 years who presented at outpatient clinics within 48 hours after the onset of influenza-like symptoms (such as fever, sore throat, cough, sneeze, or general fatigue) were screened by influenza rapid diagnostic test (RDT) kits. All patients who tested positive for influenza A or B were enrolled in the study. After obtaining written informed consent from the patients or their guardians, the clinicians prescribed one of the four NAIs. Selection of the drugs was left to the discretion of the clinicians and patients. The drugs were prescribed as standard of care with no intervention from the study authors. Drug dosages followed the standard prescription course recommended in Japan. Oseltamivir was administered orally for 5 days (2×75 mg/day for adults and children weighing ≥37.5 kg and 2 mg/kg/day for children weighing <37.5 kg). Zanamivir was administered by inhalation daily for 5 days (2×10 mg/day for adults and children ≥5 years old). Laninamivir was administered by inhalation (adults and children aged ≥10 years, as a single dose of 40 mg; and to children <10 years old, as a single dose of 20 mg). Peramivir was intravenously infused once (adults at a dose of 300 mg; children at 10 mg based on 1 kg body weight to a maximum of 600 mg, over a period of 15–30 min) [2].

Clinicians recorded patients’ age, sex, vaccination status, time from disease onset to the first clinic visit, body temperature at the clinic visit, and prescribed NAIs. Patients or guardians recorded the patients’ body temperature 3 times daily (morning, noon, and evening) until defervescence (<37.5 °C), for a maximum of 8 days at home.

The exclusion criteria from the analysis were as follows: patients identified with undifferentiated type/subtype; samples were positive for two or more influenza type/subtype; patients did not record their own temperature at home; or body temperature was <37.5 °C throughout the observation period.

Ethical approval for the study was obtained from the Ethics Committee at Niigata University (#1347).

### Detection of influenza virus

Nasopharyngeal swabs or nasal discharges were obtained from the patients at the first clinic visit and stored into virus transport media. Samples were kept frozen at -20 °C until transport to Niigata University for further viral examinations. Specimens were inoculated onto Madin-Darby canine kidney cells [19, 20]. Samples with a positive cytopathic effect were typed and subtyped using the cycling probe real-time polymerase chain reaction (PCR) method, which can identify influenza A/H1N1pdm09, A/H3N2, B/Victoria, or B/Yamagata [20]. The assay can simultaneously detect the presence of H275Y mutation in neuraminidase protein for A/H1N1pdm09 viruses [21]. In addition, susceptibility testing of A/H1N1pdm09 influenza viruses was performed using the neuraminidase inhibition assay as previously reported, against oseltamivir, zanamivir, laninamivir, and peramivir, using 2′-(4-methylumbelliferyl)-a-D-N-acetylneuraminic acid (MUNANA) to validate the results of the cycling probe real-time PCR assay [22]. The value was determined by the 50% inhibitory concentration (IC_50_) [22].

### Evaluation of fever duration

Fever duration was measured in hours, from the start of treatment until the patient reached the first point of 3 consecutive measurements <37.5 °C. If the patient had recurrent fever after 3 consecutive measurements <37.5 °C, the fever duration continued to be measured until final defervescence (regardless of the afebrile period), to prevent the influence of antipyretics. Usage of antipyretics was recorded in the patients’ home records, but this information was not used in this study because it did not affect the analyses.

### Statistical analysis

Demographic details such as sex, age, body temperature at the clinic visit, time from onset to the first clinic visit, and vaccination status were extracted from patients’ records. Each variable was tabulated by NAI groups (oseltamivir, zanamivir, laninamivir, and peramivir) and by type/subtype. For our analysis, the B/Victoria and B/Yamagata groups in the influenza B virus group were integrated into one group. For univariate analysis, analysis of variance (ANOVA) was used to compare the average of continuous variables. Fisher’s exact test was used to examine categorical variables. To estimate the bias of selecting NAIs by virus type/subtype and season, we calculated both the proportion of each virus for each treatment group as well as the proportion of each treatment group for each season. The average fever duration was evaluated by t-test if the category was two. ANOVA was used if categories were three and higher, and Bonferroni corrected t-test was employed as a post-hoc test between each pair. Categories used for evaluation were as follows: type/subtype (A/H1N1pdm09, A/H3N2, or B), age groups (0 to 5, 6 to 9, or 10 to 19 years), body temperature at clinic visit (<38.5 °C or ≥38.5 °C), time from onset to the first clinic visit (0–24 hours, or 24–48 hours), vaccination status (vaccinated or unvaccinated), and prescribed NAIs (oseltamivir, zanamivir, laninamivir, or peramivir). In addition, Kaplan-Meier method was employed to evaluate the alleviation of fever, and statistically tested by a log-rank test. Any subset with a sample size <5 was excluded from the Kaplan-Meier method analysis.

For multivariable analysis, we used Cox proportional-hazard model to evaluate the duration of fever from the first dose of the NAI to fever alleviation <37.5 °C. To assess the hazard ratio (HR), fever duration (hours) was set as the dependent variable. The following were the independent variables: NAIs treatment (zanamivir, laninamivir, or peramivir based on oseltamivir), type/subtype (A/H3N2, or B based on A/H1N1pdm09), age group (6 to 9 years, or 10 to 19 years, compared with 0 to 5 years), vaccination status (vaccinated compared with unvaccinated), time from the onset to the clinic visit (24–48 hours compared with 0–24 hours), and body temperature at the clinic visit (continuous variables).

Additionally, we conducted a sub-group analysis based on age group, in order to evaluate the relationship between a younger age and the low effectiveness of laninamivir, which is a single dose inhalation drug. ANOVA was used for this analysis.

We performed further analysis to compare fever duration after oseltamivir treatment between the H275Y mutated (oseltamivir-resistant) A/H1N1pdm09 group and the oseltamivir-sensitive group. Since the patients with H275Y mutated virus treated by oseltamivir were all aged 5 or younger, this sub-group analysis was done for those in that age group.

*P* values <0.05 were considered statistically significant. All statistical analyses were performed using EZR (Saitama Medical Center, Jichi Medical University, Saitama, Japan) [23].

## Results

### Patients’ characteristics

**Fig 1:**
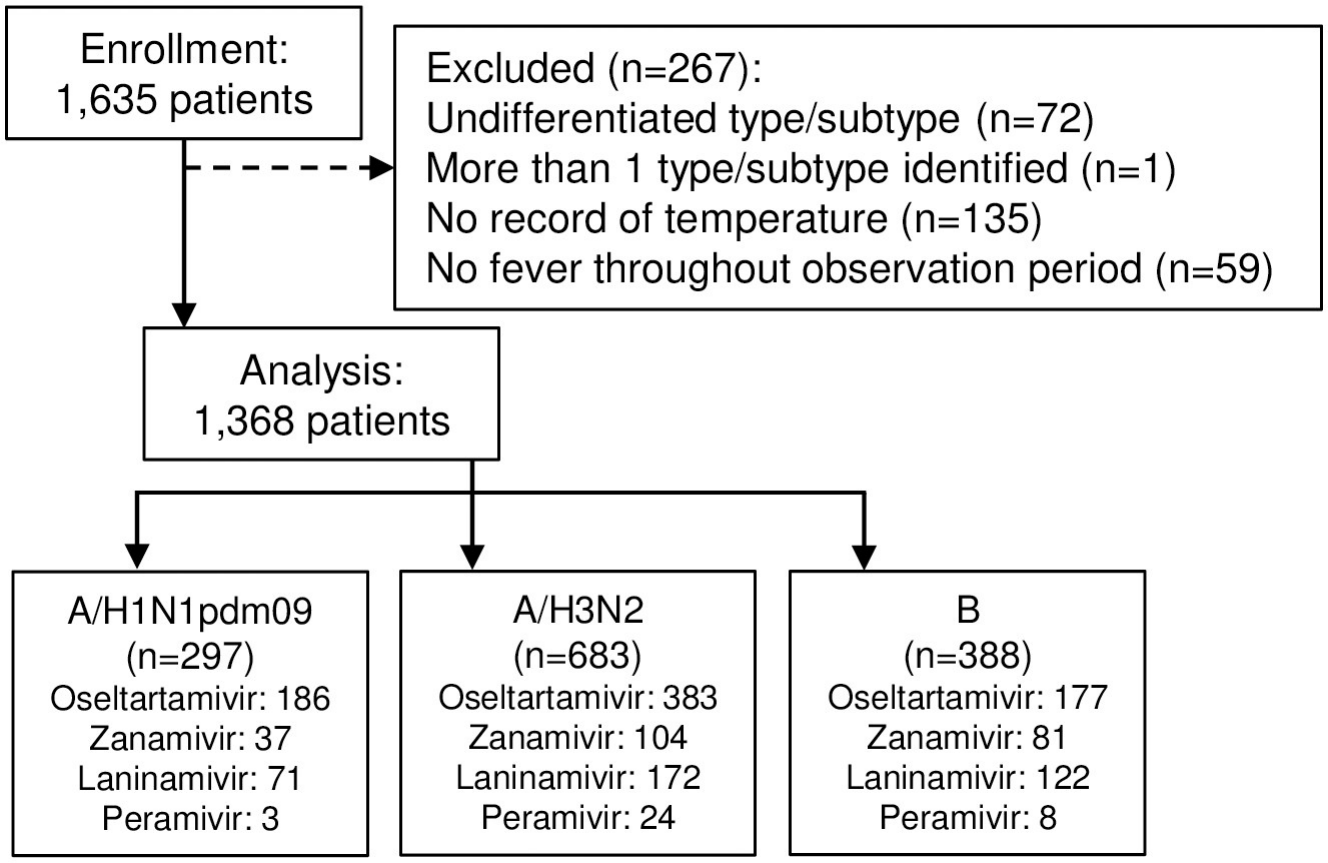
Flow of participants through the study. A total of 1,635 patients were enrolled. Patients identified with undifferentiated type/subtype (n = 72), more than 1 type/subtype virus (n = 1), no record of temperature (n = 135), and no fever throughout the observation period (n = 59) were excluded. A total of 1,368 patients were analyzed.

A total of 1,635 patients were enrolled. After excluding patients that did not meet the inclusion criteria, a total of 1,368 patients, through consecutive four influenza seasons from 2012–2013 to 2015–2016, were analyzed (Fig 1). Among them, 297 (21.7%), 683 (49.9%), and 388 (28.4%) were diagnosed with A/H1N1pdm09, A/H3N2, and B, respectively (Table 1). Influenza B was further divided into Victoria (n = 133) and Yamagata (n = 255), but they were aggregated into one group in our analysis. Most of the patients (74.3%) were aged <10 years. About half (48.8%) had body temperature of ≥38.5 °C at the first clinic visit. The vast majority of the patients (74.2%) visited the clinic within 24 hours after the onset of symptoms. Slightly over half of the patients (56.3%) had received influenza vaccine in preparation for the influenza season. Roughly half of the patients (54.5%) were treated with oseltamivir. Others were prescribed with laninamivir (26.7%), zanamivir (16.2%), and peramivir (2.6%).

**Table 1:** Baseline characteristics.

Category	n (%)
Type/subtype	
A/H1N1pdm09	297 (21.7)
A/H3N2	683 (49.9)
B/Victoria	133 (9.7)
B/Yamagata	255 (18.6)
Age group	
0–5 years	466 (34.1)
6–9 years	550 (40.2)
10–19 years	352 (25.7)
Body temperature at clinic visit	
<38.5 °C	701 (51.2)
≥38.5 °C	667 (48.8)
Time from onset to the first clinic visit	
0–24 hours	1015 (74.2)
24–48 hours	353 (25.8)
Vaccination status ^a^	
Unvaccinated	581 (43.7)
Vaccinated	749 (56.3)
Treatment	
Oseltamivir	746 (54.5)
Zanamivir	222 (16.2)
Laninamivir	365 (26.7)
Peramivir	35 (2.6)

^a^ Thirty-eight missing data because of unknown vaccination status.

Baseline characteristics were further stratified by influenza types/subtypes and treatment groups. For A/H1N1pdm09, the average age for oseltamivir treatment group (6.2 ± 7.3 years) was younger than that for zanamivir (10.1 ± 2.7) and laninamivir (9.3 ± 2.2 years) treatment groups (Table 2). In Japan, oseltamivir was not recommended for use among teenagers because of the possibility of abnormal behaviors [2]. The average age for the peramivir treatment group (6.0 ± 4.4 years) was the lowest, because this drug was more used for younger children when oral or inhalation medication was difficult. Although the number of patients in the peramivir group was low and the statistical power was lacking, we retained the peramivir treatment group in our analysis because the clinical effectiveness of peramivir in outpatients has rarely been reported [24, 25]. The patient characteristics for influenza A/H3N2 and B were similar to those for A/H1N1pdm09, for all categories.

**Table 2:** Patients’ characteristics by treatment groups and type/subtype.

Type/subtype of influenza	Value				
	Oseltamivir treatment group	Zanamivir treatment group	Laninamivir treatment group	Peramivir treatment group	*P* value
**A/H1N1pdm09 (N = 297)**					
N	186	37	71	3	
Male/female	101/85	18/19	38/33	2/1	0.817^a^
Age (years), mean ± SD	4.7 ± 2.4	10.1 ± 2.7	9.3 ± 2.2	6.0 ± 4.4	<0.001^a^^,^ ^b^
Body temperature at the clinic visit (°C), mean ± SD	38.6 ± 0.8	38.6 ± 0.7	38.5 ± 0.7	38.3 ± 1.8	0.256^a^
Time from onset to the first clinic visit <24 hours (%)	81.2	78.4	83.1	33.0	0.838^a^
Vaccination (%)	47.5^c^	56.8	58.6^c^	66.7	0.236^a^
**A/H3N2 (N = 683)**					
N	383	104	172	24	
Male/female	225/158	45/59	97/75	14/10	0.046
Age (years), mean ± SD	4.9 ± 2.3	10.4 ± 2.9	10.4 ± 2.4	5.1 ± 4.4	<0.001^d^
Body temperature at the clinic visit (°C), mean ± SD	38.4 ± 0.8	38.4 ± 0.8	38.3 ± 0.7	38.6 ± 0.7	0.026^e^
Time from onset to the first clinic visit <24 (%)	66.6	73.1	67.4	75.0	0.566
Vaccination (%)	56.1^c^	44.6^c^	55.1^c^	65.2^c^	0.004^f^
**B (N = 388)**					
N	177	81	122	8	
Male/female	93/84	45/36	64/58	2/6	0.458
Age (years), mean ± SD	5.6 ± 2.0	9.5 ± 2.3	9.8 ± 2.2	7.5 ± 4.1	<0.001^g^
Body temperature at the clinic visit (°C), mean ± SD	38.3 ± 0.8	38.5 ± 0.6	38.3 ± 0.7	38.8 ± 0.7	0.069
Time from onset to the first clinic visit <24 (%)	78.0	82.7	82.0	62.5	0.413
Vaccination (%)	59.9 ^c^	58.8 ^c^	49.1 ^c^	37.5	0.202

SD: standard deviation

^a^ The group with a sample size <5 was excluded from analysis.

^b^ Multiple comparison by Bonferroni correction showed that the mean age for the oseltamivir treatment group was lower than that for the zanamivir and laninamivir treatment groups (p <0.001).

^c^ There were some missing data.

^d^ Multiple comparison by Bonferroni correction showed that the mean age for the oseltamivir treatment group was lower than that for the zanamivir and laninamivir treatment groups (p <0.001). The mean age for the peramivir treatment group was lower than that for the zanamivir and laninamivir treatment group (p <0.001).

^e^ Multiple comparison by Bonferroni correction showed that the body temperature for the oseltamivir treatment group was higher than that for the laninamivir treatment group (p = 0.042).

^f^ Multiple comparison by Bonferroni correction showed that the proportion of vaccinated patients was higher in oseltamivir treatment group than that in the zanamivir treatment group (p = 0.005).

^g^ Multiple comparison by Bonferroni correction showed that the mean age for the oseltamivir treatment group was lower than that for the zanamivir (p<0.001) and laninamivir treatment groups (p <0.001). The mean age for the peramivir treatment group was lower than that for the laninamivir group (p = 0.025).

**Fig 2:**
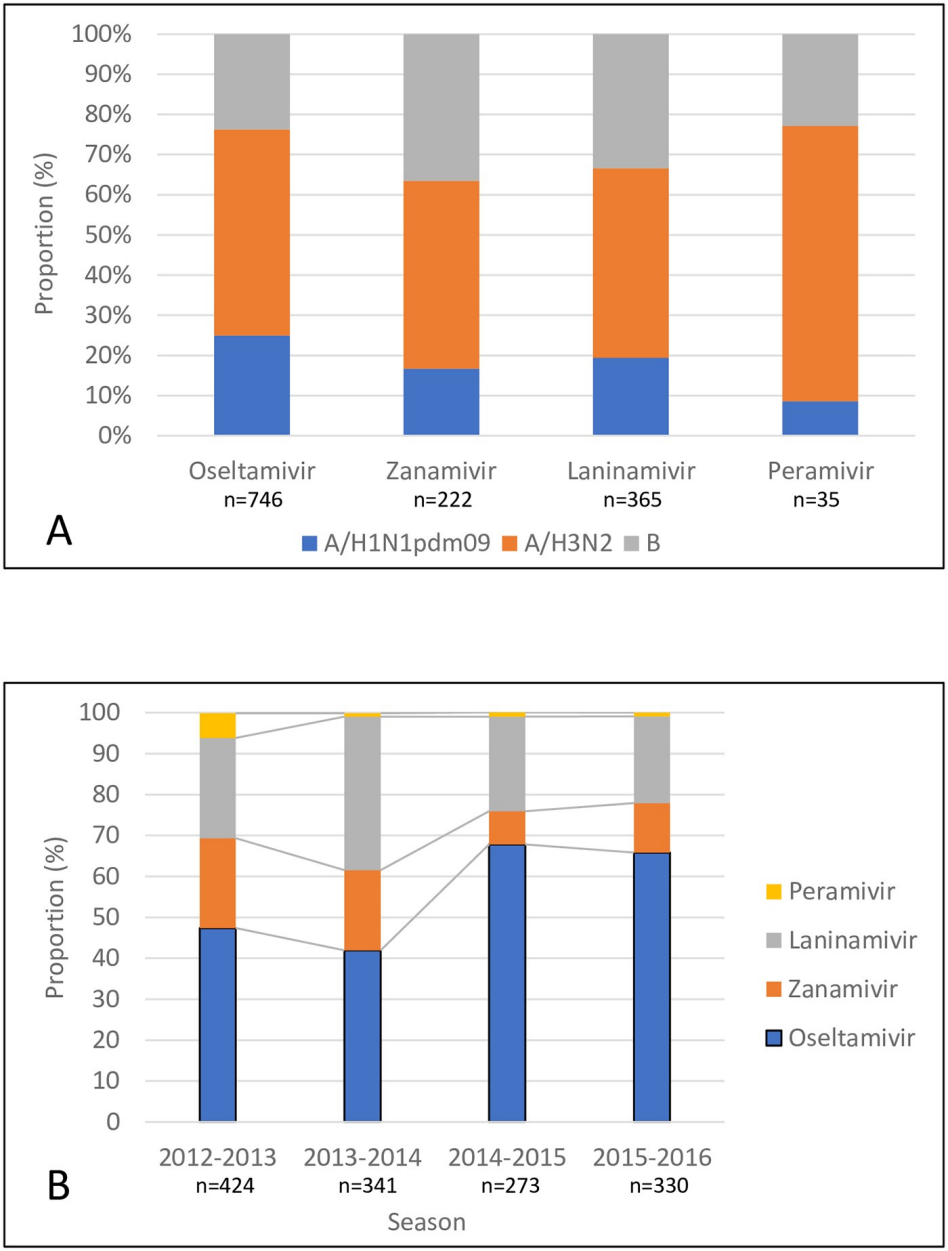
Variation of type/subtype of influenza virus in each NAI treatment group and selection of NAIs by season. A: Proportion of type/subtype of influenza virus in each NAI treatment group. B: Proportion of each NAI treatment group by season. NAI, neuraminidase inhibitors.

The proportion of various types/subtypes of influenza virus in each NAI treatment group is shown in Fig 2A. Among all treatment groups, the A/H3N2 group had the highest proportion of the influenza virus (47–69%). The influenza B proportion was higher in the zanamivir (33%) or laninamivir (36%) treatment groups than in the oseltamivir (24%) or peramivir (23%) treatment groups. In Japan, Kawai et al. reported that the fever duration for zanamivir was shorter than for oseltamivir in influenza B patients [26]. Therefore, Japanese physicians may prescribe zanamivir more frequently for influenza B than for influenza A.

Throughout all seasons, the oseltamivir treatment group had the highest proportion (42–68%) (Fig 2B).

### Univariate analyses of fever duration by the different characteristics

ANOVA and Bonferroni corrected t-test showed that the average duration of fever was significantly longer for influenza B than for A/H1N1pdm09 and A/H3N2 (p<0.001) (Table 3 and S1 Fig). The fever duration for the A/H1N1pdm09 group was longer than that for the A/H3N2 group (p = 0.028). Patients aged 0 to 5 years had longer fever duration than those aged 10 to 19 years (p<0.001). Patients with body temperature ≥38.5 °C at the clinic visit showed longer fever duration than those with temperature <38.5 °C (p = 0.004). Patients who visited within 24 hours of the onset of symptoms, compared to those who visited 24–48 hours after, had longer fever duration (p<0.001); the unvaccinated group had longer duration than the vaccinated (p = 0.029). However, no significant difference was seen among the four NAI groups.

**Table 3:** Univariate analyses of the mean fever duration after NAI treatment, according to different characteristics.

Characteristics			Mean (hours)	SD	*P* value*
Type/subtype	A/H1N1pdm09	n = 297	38.7	24.7	<0.001^a^
	A/H3N2	n = 683	34.2	21.4	
	B	n = 388	50.1	30.0	
Age group	0–5 years	n = 466	42.8	28.9	<0.001^b^
	6–9 years	n = 550	39.5	24.0	
	10–19 years	n = 352	35.9	23.2	
Body temperature at clinic visit	<38.5 °C	n = 701	37.8	25.4	0.004
	≥38.5 °C	n = 667	41.8	25.9	
Time from onset to the first clinic visit	0–24 hours	n = 1,015	41.2	26.3	<0.001
	24–48 hours	n = 353	35.5	23.6	
Vaccination status	Unvaccinated	n = 581	41.4	27.1	0.029
	Vaccinated	n = 749	38.3	24.5	
Treatment	Oseltamivir	n = 746	40.1	26.0	0.850
	Zanamivir	n = 222	38.5	24.2	
	Laninamivir	n = 365	39.8	25.5	
	Peramivir	n = 35	38.3	31.1	

NAI: neuraminidase inhibitor, SD: standard deviation

*Average durations of fever were compared among the different characteristics using ANOVA.

^a^ Multiple comparison adjusted by Bonferroni correction showed that the fever duration for both subtype A/H1N1pdm09 and A/H3N2 groups were shorter than that for B groups (p<0.001). The fever duration for A/H1N1pdm09 group was longer than that for the A/H3N2 group (p = 0.028).

^b^ Multiple comparison adjusted by Bonferroni correction showed that the fever duration for those aged 0–5 years was longer than for those aged 10–19 years (p<0.001).

### Univariate analysis of fever duration for four NAIs by each type/subtype

**Fig 3:**
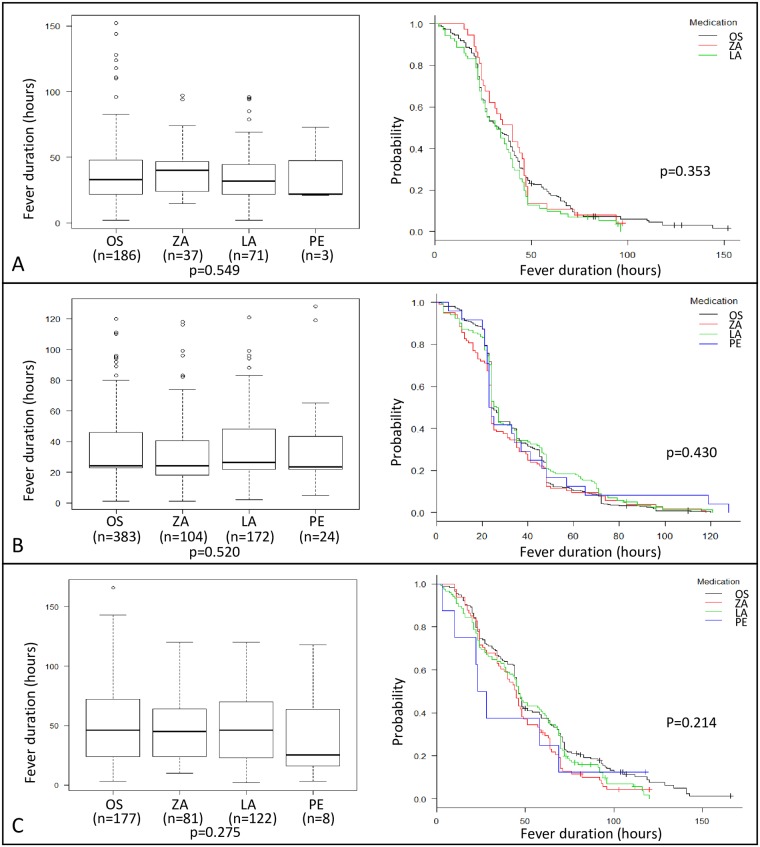
The comparison of fever duration by the four NAIs by type/subtype group. A: A/H1N1pdm09 group. B: A/H3N2 group. C: influenza B group. Left: Boxplots of fever duration for the four NAIs tested by ANOVA. The bold line is the average, while the box shows the interquartile range. *P*-value at the bottom was obtained from ANOVA. Right: Kaplan-Meyer method of fever duration. Black line: oseltamivir, red line: zanamivir, green line: laninamivir, and blue line: peramivir. *P*-value from log-rank analysis is indicated in the graph. The group with a sample size <5 was excluded from analysis. OS: oseltamivir, ZA: zanamivir, LA: laninamivir, PE: peramivir, NAI: neuraminidase inhibitor.

When stratified by type and subtype, the average fever duration for A/H1N1pdm09 (p = 0.549), A/H3N2 (p = 0.520), and influenza B (p = 0.275) was similar among the four NAIs (Fig 3). The results of the Kaplan-Meier method supported the ANOVA results (Fig 3).

### Multivariable analysis of fever duration

We conducted multivariable analysis using Cox-proportional hazard model to evaluate factors associated with the alleviation of fever. HR <1 indicated prolonged fever alleviation for the variable. Treatment (laninamivir [HR = 0.78, p = 0.006] compared to oseltamivir); type/subtype (B [HR = 0.58, p<0.001] compared to A/H1N1pdm09 infection); and higher body temperature at the clinic visit (HR = 0.88, p<0.001) were significantly associated with longer fever duration (Table 4). However, compared to age 0 to 5 years, increasing age (6 to 9 years [HR = 1.31, p<0.001] and 10 to 19 years [HR = 1.67, p<0.001]) showed shorter fever alleviation durations. No association was shown with vaccination status and time from the onset to clinic.

**Table 4:** Cox proportional hazards model of fever duration.

		HR	95% CI	*P* value
Treatment	Oseltamivir	1.00		
	Zanamivir	0.87	0.72–1.06	0.171
	Laninamivir	0.78	0.65–0.93	0.006
	Peramivir	1.05	0.73–1.49	0.798
Type/subtype	A/H1N1pdm09	1.00		
	A/H3N2	1.09	0.94–1.25	0.259
	B	0.58	0.49–0.68	<0.001
Age	0 to 5 years	1.00		
	6 to 9 years	1.31	1.14–1.52	<0.001
	10 to 19 years	1.65	1.34–2.04	<0.001
Vaccination	Unvaccinated	1.00		
	Vaccinated	1.09	0.98–1.23	0.120
Time from onset to the first clinic visit	0–24 hours	1.00		
	24–48 hours	1.08	0.95–1.23	0.234
Body temperature at the clinic visit		0.87	0.81–0.94	<0.001

Hazard ratio <1 indicates longer fever duration compared to that of the reference group. HR: hazard ratio. CI: confidential interval.

### Sub-group analysis of fever duration for four NAIs by age group

In the 0 to 5 years age group, the average fever duration for laninamivir (75.8 hours) was longer than that for oseltamivir (42.2 hours) (p = 0.028) (Table 5). In the 6 to 9 years age group, the average fever duration for laninamivir (42.9 hours) tended to be longer than that for oseltamivir (37.3 hours), but the difference was not significant (p = 0.120). In the 10 to 19 years age group, the average fever duration for four NAIs was not significantly different.

**Table 5:** Sub-group analysis of fever duration for four NAIs by age group.

	N	Average	SD	*P* value
0 to 5 years				
Oseltamivir	439	42.2	28.3	0.040^a^
Zanamivir	5	45.4	40.9	
Laninamivir	6	75.8	34.6	
Peramivir	16	46.3	34.1	
6 to 9 years				
Oseltamivir	299	37.3	22.2	0.042^b^
Zanamivir	91	42.3	24.5	
Laninamivir	148	42.9	26.0	
Peramivir	12	31.3	31.5	
10 to 19 years				
Oseltamivir	8	29.8	12.6	0.813
Zanamivir	126	35.4	23.0	
Laninamivir	211	36.6	23.8	
Peramivir	7	32.3	20.9	

^a^ Multiple comparison adjusted by Bonferroni correction showed that the fever duration for laninamivir was longer than that for oseltamivir group (p = 0.028).

^b^ Multiple comparison adjusted by Bonferroni correction showed that the fever duration for laninamivir tended to be longer than that for oseltamivir group (p = 0.120).

### Fever duration after oseltamivir treatment for H275Y mutated A/H1N1pdm09 virus infection

**Fig 4:**
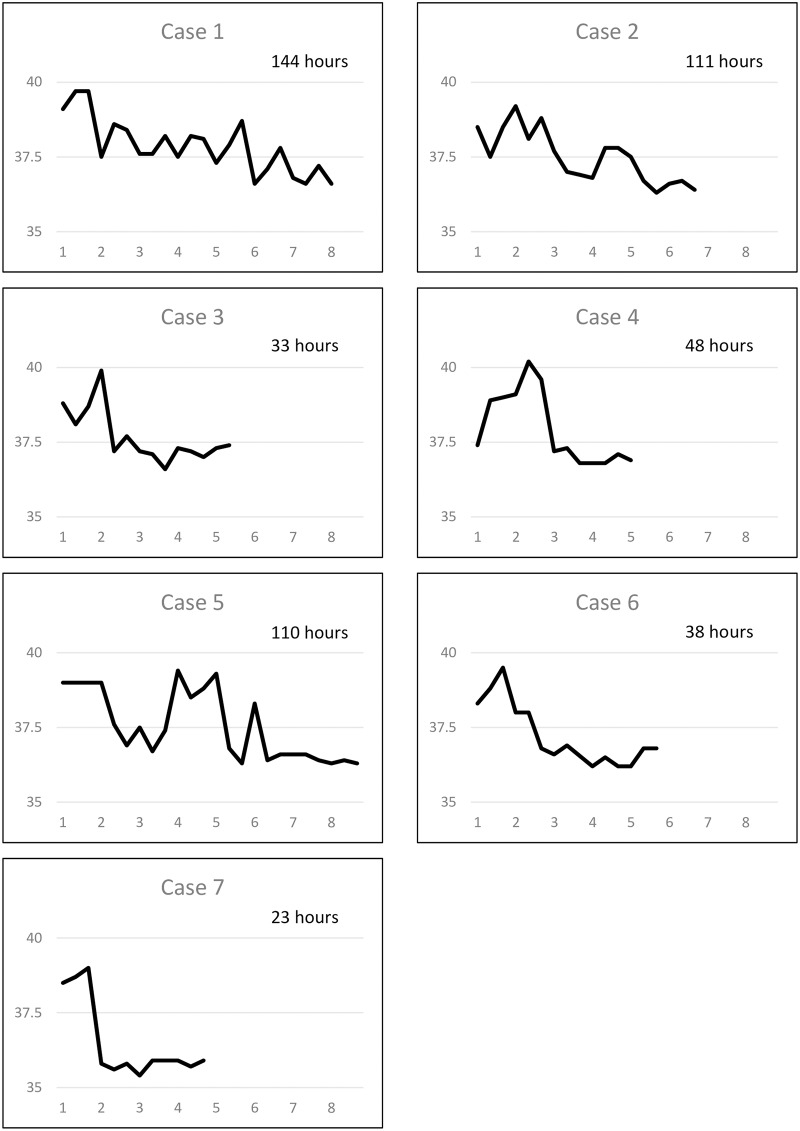
Temperature curve of the patients infected with influenza A/H1N1pdm09 with the H275Y mutation. Patients in cases 1–5 received oseltamivir. The patient in case 6 received laninamivir and that in case 7 received zanamivir. Fever duration is shown in the right upper field in each box.

**Fig 5:**
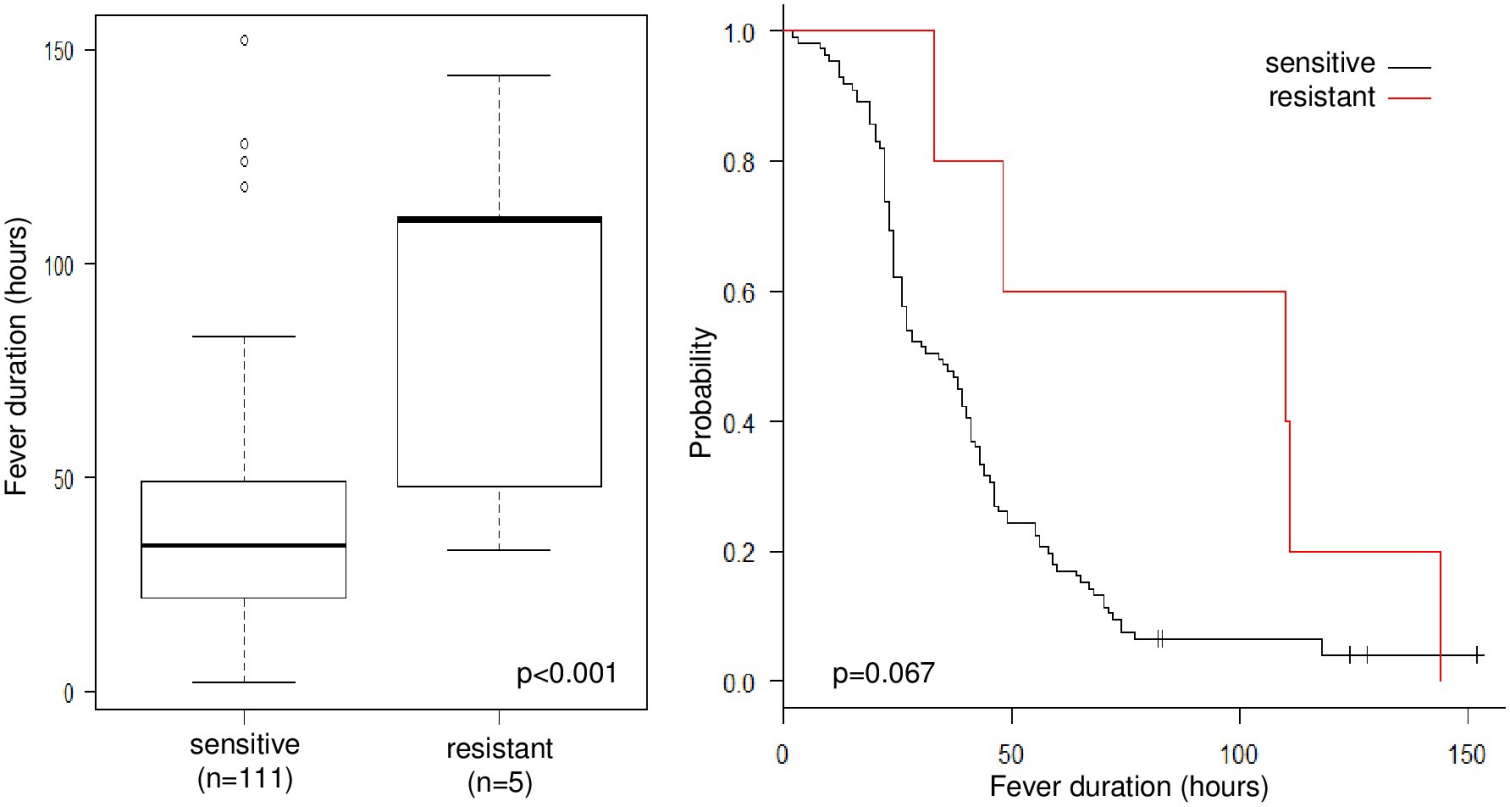
Fever duration comparison between A/H1N1pdm09 oseltamivir-resistant and oseltamivir-sensitive patient groups aged 0 to 5 years. Left: Boxplots of t-test for the average fever duration. The bold line is the average value; the box shows the interquartile range. Right: Kaplan-Meier method for fever duration. Black line: oseltamivir-sensitive; red line: oseltamivir-resistant.

We detected seven cases of A/H1N1pdm09 with H275Y mutation in neuraminidase protein, which conferred resistance to oseltamivir and peramivir. The IC_50_ value against oseltamivir for these 7 isolates ranged from 291.77 to 395.24 nM (S1 Table). Five cases were treated with oseltamivir, one with laninamivir, and another with zanamivir (Fig 4). In patients who were treated with laninamivir or zanamivir, fever was alleviated within 48 hours. Fever in 2 (40%) of the patients that were treated with oseltamivir was also alleviated within 48 hours; however, the remaining 3 patients (60%) had prolonged fever, which continued for more than 100 hours. In oseltamivir-treated patients who were infected with A/H1N1pdm09, the average fever duration was longer in patients (0–5 years) infected with oseltamivir-resistant viruses (n = 5, 89 ± 47 hours) than oseltamivir-sensitive viruses (n = 111, 40 ± 27 hours, p<0.001); (Fig 5). However, the log-rank test for Kaplan-Meier method was marginally significant (p = 0.067). We did not carry out multivariable analysis for this sub-group due to the small sample size of H275Y cases.

## Discussion

In this study we compared the clinical effectiveness of four NAIs (for children and adults aged <20 years) against laboratory confirmed influenza A/H1N1pdm09, A/H3N2, and B. We found that influenza B infections, laninamivir treatment, younger age (0–5 years), and higher body temperature at clinic visit were associated with longer fever duration after the commencement of treatment, in multivariable analysis. We also found that fever duration in young children (0–5 years) infected with H275Y mutated influenza A/H1N1pdm09 virus and treated with oseltamivir was prolonged, compared to those with oseltamivir-sensitive virus infections. Our results revealed important information regarding the clinical effectiveness, especially of laninamivir and peramivir that are licensed for outpatients only in Japan, and also regarding oseltamivir-resistant viruses for which only limited clinical information is available.

Our results also showed that fever alleviation in influenza B infections was delayed roughly by half a day (~ 12 hours) compared to influenza A. Multivariable analysis also showed that influenza B infections was an independent risk factor for prolonged fever regardless of the treatment used among patients aged <20 years. In several studies, longer fever duration for influenza B, ranging from 13 to 36 hours after NAI treatment, have been reported [4, 5, 27, 28]. The reason for poor outcomes for influenza B than for influenza A remains unclear. Influenza B is reported to have a lower susceptibility to NAIs than does influenza A (2 to 16-fold changes) [29]. The virus re-isolation rate is significantly higher for influenza B than for influenza A after 4–6 days of oseltamivir therapy [30]. These might have caused the prolonged fever duration in patients with influenza B.

In our study, laninamivir treated group had significantly longer fever duration (HR, 0.78, p<0.01) compared to oseltamivir group in multivariable analysis. Laninamivir is a single dose, long-acting drug that is administered by inhalation, while oseltamivir and zanamivir are administered twice daily for 5 days. Ishiguro et al. reported that fever duration in laninamivir-treated influenza A or B infections was longer compared to oseltamivir or peramivir-treated infections among outpatients aged 0–18 years using Cox-proportional hazard model [24]. Koseki et al. reported that the frequency of biphasic fever was significantly higher in laninamivir-treated patients than in zanamivir-treated patients, although the fever duration for zanamivir and laninamivir was not significantly different among patients aged 5 to 18 years [31]. Hikita et al. reported that the median fever duration after treatment with laninamivir of 3 days, was significantly longer by 1 day for peramivir in influenza B-infected patients aged 5 to 18 years (P <0.01) [32]. Sugaya et al. reported a significantly shorter fever duration (0.75 to 1.10 day) in patients administerd peramivir than in those administered laninamivir (P<0.05) among influenza A/H3N2 or A/H1N1pdm09 infections [33]. Ishiguro et al. suggested the possibility that an inadequate inhalation of the single dose laninamivir influenced the longer fever duration in patients aged 5 to 9 years than those treated with other NAIs [24]. Koseki et al. also suggested that incomplete inhalation of the single dose of laninamivir, especially in young children, could explain the increase in frequency of biphasic fever in the laninamivir treated group compared with zanamivir treated group [31]. Our study also showed that the average fever duration was longer in laninamivir treated group than in those who were treated by oseltamivir among the younger age. This might have been caused by inadequate inhalation in the younger age group. Nevertheless, variable results have been reported for the four NAIs [24, 25, 34–36]. Therefore, there is need for continuing evaluation of the clinical effects of the four NAIs.

Our study showed that a younger age was a factor of prolonged fever. Past studies repeatedly showed that younger children had longer fever duration than older children or adults [5, 24, 31, 37, 38]. Prolonged fever in young children may be due to the longer virus shedding in younger patients than older patients [33, 39]. It is also known that fever is often prolonged in immunocompromised patients who shed virus longer than immunocompetent patients [40]. Prolonged viral shedding in children could have resulted from low immunity due to the less experience of infections [24, 41].

Patients with higher body temperature at the clinic visit had longer fever duration in this study. Kawai et al. reported similar results that patients with high peak body temperature had longer fever duration [42]. Kaiser et al. reported that viral titers correlated positively with symptoms and temperature, and that cytokine level, for such as interleukin 6 and tumor necrosis factor alpha, also correlated positively with temperature [43]. Prolonged fever caused by high body temperature at the clinic visit may have resulted from higher viral titer and higher cytokine levels.

In univariate analysis, unvaccinated status was associated with prolonger fever, but this was not significant in multivariable analysis. Tochino et al. reported longer fever duration among vaccinated than among unvaccinated patients in univariate but not in multivariable analysis [44]. In this previous study, the vaccinated patients were younger; therefore, being younger vaccinated patients might have led to the longer fever duration. In our study, unvaccinated patients were older than the vaccinated patients (8.9 versus 7.7 years, p<0.01); however, the unvaccinated patients had higher temperature at the clinic visit that might have resulted in the prolonger fever duration shown in univariate analysis (38.5 versus 38.3 °C, p<0.01) that was offset in multivariable analysis. As suggested by Tochino et al., differences in medical care-seeking behavior may exist in vaccinated patients with low grade fever and/or mild symptoms who did not go to the hospital.

Current study showed that the fever duration was longer in the oseltamivir-resistant group than in the oseltamivir-sensitive group among influenza A/H1N1pdm09-infected patients. Several studies reported that when patients were treated with oseltamivir, oseltamivir-resistant former influenza A/H1N1 circulating during 2007–2008 demonstrated prolonged fever compared to oseltamivir-sensitive influenza [12, 13, 45]. The Influenza Resistance Information Study showed that viral RNA was detected for longer periods in patients infected with oseltamivir-resistant influenza A/H1N1pdm09 and A/H3N2 viruses, although these patients exhibited a shorter duration of symptoms compared to those infected with oseltamivir-sensitive influenza [15]. We previously reported five cases of oseltamivir and peramivir-resistant influenza A/H1N1pdm09 in 2015/2016 season [19]. In all these patients, following treatment with oseltamivir or peramivir, the fever subsided within 48 hours of the start of treatment [19]. Only the two child cases from the previous report were included in the current study. In the former A/H1N1 infection, younger children showed prolonged fever duration, which was not reported among the adults [45]. Exclusion of adult patients in this study may have led to the prolonged fever duration in the H275Y cases.

Our study has some limitations. Because this was an observational study, the selection of NAIs was left to the physician’s discretion. The sample size of patients receiving peramivir was not large, hence accurate statistical analysis could not be conducted; thus, caution is necessary when interpreting the results. Because peramivir is an injectable drug, it is easy to administer to patients who cannot take oral medicines or inhalants, such as seriously ill patients or young infants. Moreover, because oseltamivir is not recommended for use in those aged 10 to 19 years (because of abnormal behavior) by the order of the Ministry of Health, Labor, and Welfare [2], this skewed our patients’ age to the younger side, in the oseltamivir treatment group than that in the zanamivir or laninamivir groups. Since zanamivir- and laninamivir-treated patients had similar mean age, the results between zanamivir and laninamivir were comparable. Nevertheless, we used the multivariable analysis to adjust for those age differences. The viral load and severity of illness data were not evaluated in this study. We collected the second samples after 3–7 days of the first visit to measure viral load shift by the treatment, but the number of samples are too less, compared to those who clinical records were obtained. Thus, we decided not to include viral load analysis in this study, but in the future study. For the diseases severity, our sample size was too small to evaluate the frequency of severity such as admission rates or mortality with enough statistical power on study group that was otherwise healthy outpatients, so we excluded it from our analysis. In the comparison between oseltamivir-resistant and oseltamivir-sensitive influenza, the sample size of oseltamivir-resistant influenza patients was also limited.

In conclusion, laninamivir treatment showed longer fever durations among the four NAIs. Fever duration was prolonged in children treated with oseltamivir for A/H1N1pdm09 harboring H275Y mutation. Thus, our study can serve as comparative evidence to assess the clinical effectiveness of another class of drug (cap-dependent endonuclease inhibitor) recently approved in Japan and United States [46], which can be an effective therapeutic option when administered alone or in combination [47].

## Supporting information

S1 Fig Univariate analysis of the fever duration by each characteristic. A: Type/subtype (A1: A/H1N1pdm09; A3: A/H3N2; B: influenza B), B: Age group. C: Body temperature at the clinic visit. D: Time from disease onset to the first clinic visit. E: Vaccine status. F: Treatment (OS: oseltamivir, ZA: zanamivir, LA: laninamivir, PE: peramivir). Left: Boxplots of ANOVA or t test of the average fever duration. Bold line is the mean; the box shows the interquartile range. Right: Kaplan-Meier method of analysis for the fever duration.
(PDF)

S1 Table The 50% inhibitory concentrations and administered medications in patients infected with H275Y mutated influenza A/H1N1pdm09 virus. (XLSX)
