# Supplementary figures and images for: Effectiveness of four types of neuraminidase inhibitors approved in Japan for the treatment of influenza
Source: PLoS One. 2019 Nov 7;14(11):e0224683. doi: 10.1371/journal.pone.0224683 (PMC6837752; doi:10.1371/journal.pone.0224683)

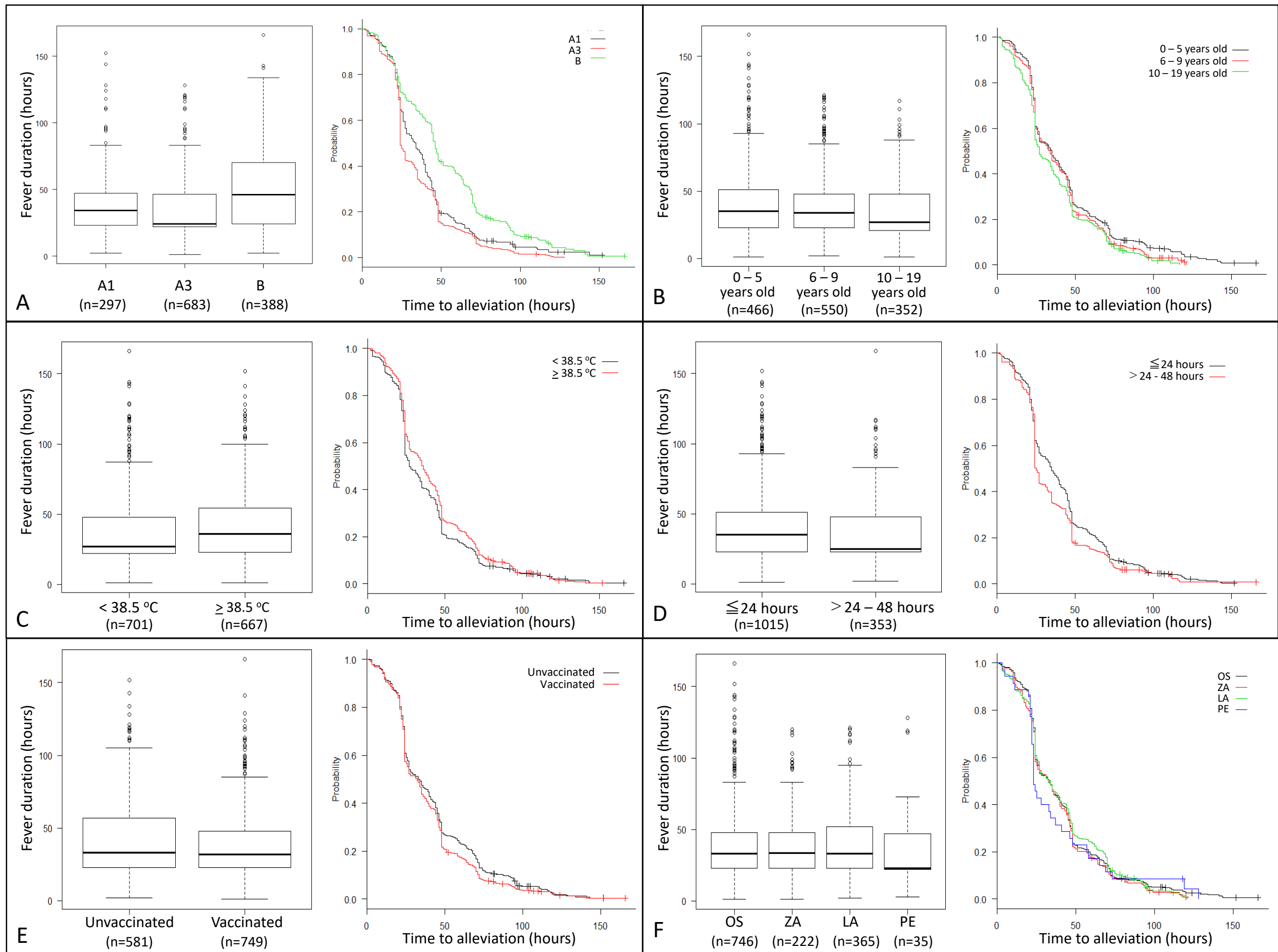

Supplement: S1 Fig — A: Type/subtype (A1: A/H1N1pdm09; A3: A/H3N2; B: influenza B), B: Age group. C: Body temperature at the clinic visit. D: Time from disease onset to the first clinic visit. E: Vaccine status. F: Treatment (OS: oseltamivir, ZA: zanamivir, LA: laninamivir, PE: peramivir). Left: Boxplots of ANOVA or t test of the average fever duration. Bold line is the mean; the box shows the interquartile range. Right: Kaplan-Meier method of analysis for the fever duration. (PDF) [file pone.0224683.s001.pdf]
